# Radiological characterization related to lithology and risk assessment of bottled natural mineral water

**DOI:** 10.1007/s10661-024-13353-z

**Published:** 2024-11-05

**Authors:** Joana Martínez, Alejandra Peñalver, Jordi Riu, Carme Aguilar, Francesc Borrull

**Affiliations:** 1 Departament de Química Analítica i Química Orgànica, Universitat Rovira i Virgili, Unitat de Radioquímica Ambiental i Sanitària, Ctra. Nacional 340, Km. 1094, 43895 L’Ampolla, Tarragona, Spain; 2 Present Address: Departament de Química Analítica i Química Orgànica, Universitat Rovira i Virgili, Campus Sescelades, Building N4, Marcel·lí Domingo, 1, Tarragona, Spain

**Keywords:** Natural mineral water, Bottled, Lithology, Natural radioactivity, Risk assessment, Annual effective dose

## Abstract

The enhancement of natural radioactivity in groundwater, specifically in natural mineral water, is related to the lithological formations through which water bodies or courses pass. Although natural mineral waters are exempt from monitoring for radioactive substances according to Council Directive 2013/51/EURATOM, this study focuses on the radiological characterization of natural mineral water under Spanish Royal Decree 3/2023. The water studied was taken from Catalan aquifers with different lithological characteristics (sedimentary, metamorphic or granitic) and is sold on local markets. Moreover, radiological data on the water was correlated with its lithological origin and the health risk for different age groups was assessed. Our results showed that of the 26 natural mineral waters studied, 10 exceeded gross alpha screening value (100 mBq/L), all from granitic aquifers. Further research on natural individual radionuclides was conducted on these ten samples. ^234^U and ^238^U were at around 1100–1600 mBq/L. In addition, ^210^Pb was found in two samples, which also presented the highest ^226^Ra activity, associated with granitic bedrock and the presence of ^210^Po. The annual effective dose was 179.0 µSv/year and 145.9 µSv/year, exceeding 100 µSv/year mainly due to the contribution of ^210^Pb > ^234,238^U > ^210^Po > ^226^Ra, in this order. After assessing the lifetime cancer risk, these two samples were determined not to pose a health risk due to ingestion. Although no radiological monitoring is required for natural mineral water, further surveillance is recommendable.

## Introduction

Access to drinking water is a human right. Water must be available, safe and affordable for personal and domestic use (UN General Assembly, 2016). Generally speaking, drinking water comes from two sources: the tap and bottles. Although tap water is considered suitable for human consumption and has to meet strict quality standards, bottled water seems to be preferred by consumers worldwide, primarily for its organoleptic properties (Bouhlel et al., 2023; Yu et al., 2020). Specifically, 82% of bottled waters consumed in Europe are natural mineral water (NMW) (International Atomic Energy Agency, 2023). Spain ranks among the top five countries in Europe for bottled water consumption (Asociación Aguas Minerales de España, 2022; Natural Mineral Waters Europe, 2022), with 21% of bottled NMW volume coming from Catalonia (north-eastern Spain). This water originates from groundwater sources and is characterized by its bacteriological health, mineral content, trace elements and other components (Manteca, 2004).

NMW quality is assessed based on microbial, chemical and physical parameters. Moreover, the presence of radioactive substances in NMW, generally naturally occurring radionuclides from the ^238^U and ^232^Th decay series transferred from bedrock, must also be considered. In Spain, Royal Decree 1798/2010 (Ministerio de la Presidencia, 2011) regulates the exploitation and marketing of mineral waters and bottled spring waters for human consumption. However, unlike drinking water and spring waters, NMW is exempt from assessment for radioactive substances according to Council Directive 2013/51/EURATOM. Directive 2009/54/EC (Official Journal of the European Union, 2009) specifies that only radio-actinological properties at the source need to be established for NMW, with no regulatory quality limit set for radioactivity. Therefore, in the absence of a regulatory framework and considering that NMW is intended for human consumption, this study assessed radioactive substances within the context of Spanish drinking water legislation, namely RD 3/2023 (Ministerio de la Presidencia, 2023). The legislation specifies gross alpha and gross beta screening parametric values of 100 mBq/L and 1000 mBq/L, respectively. If these parameters are exceeded, further investigation on individual natural radionuclides (^210^Pb, ^210^Po, ^224^Ra, ^226^Ra, ^234^U, ^238^U) is necessary.

The presence of radioactive substances in NMW is influenced by the geology of the water source, among other factors such as the aquifer redox conditions, weathering, seasonal precipitation variation and water residence time (Altlkulaç et al., 2022; International Atomic Energy Agency, 2023; Tapias et al., 2022). Consequently, the composition of each NMW is unique depending on its source. Several studies worldwide have reported great variability in the activity of natural radionuclides in bottled water or drinking water from groundwater sources depending on aquifer conditions and lithology (Altlkulaç et al., 2022; Bazza et al., 2023; Borrego-Alonso et al., 2023; Chmielewska et al., 2020; Khandaker et al., 2017; Kinahan et al., 2020; Pérez-Moreno et al., 2020; Piñero-García et al., 2021; Slavchev et al., 2022; Yu et al., 2020). For example, water from areas dominated by granitic formations tends to exhibit higher gross alpha activity (Pérez-Moreno et al., 2020). In those cases, the ^210^Po content in groundwater can exceed 5 mBq/L, influenced by factors like seasonal precipitation, among others, as Slavchev et al. (2022) determined in Bulgarian drinking water. Additionally, ^222^Rn activity is also higher in granitic formations than in sedimentary or metamorphic rock aquifers. For example, ^222^Rn activity of kBq/m^3^ magnitude has been reported in Chinese and US groundwaters from granitic areas (Zhuo et al., 2001).

Although the ingestion of drinking water only contributes a small fraction of radiation to the average annual committed dose, human internal exposure must be identified and recognized. Prolonged internal exposure to natural radionuclides such as uranium, radium, lead and polonium could pose health risks. However, drinking water that results in an annual consumption of 100 µSv/year or less is not expected to give rise to detectable adverse health effects (World Health Organization, 2022). Therefore, annual committed dose evaluation is a useful tool for protecting consumers from radiation, identifying and preventing water quality problems, and ensuring compliance with current legislation.

Given these considerations, this study aims to contribute to a broader understanding of the radiological content in Catalan bottled mineral water. Specifically, it aims to determine gross alpha, gross beta, ^222^Rn and individual natural radionuclide activity in the event that these parametric values are exceeded, as well as the physicochemical parameters in 26 brands of bottled NMW in Catalonia (listed in the Official Journal of the European Union). Statistical analyses were conducted to explore the relationship between radiological composition and lithological origin. Finally, the annual effective dose for different age groups (infants, children and adults) and the risk of cancer due to ingestion of NMW was evaluated.

## Material and methods

### Study area

Catalonia is situated in the north-eastern Iberian Peninsula, covering an area of 32,108 km^2^ with over 7.566 million inhabitants (2019).

**Fig. 1:**
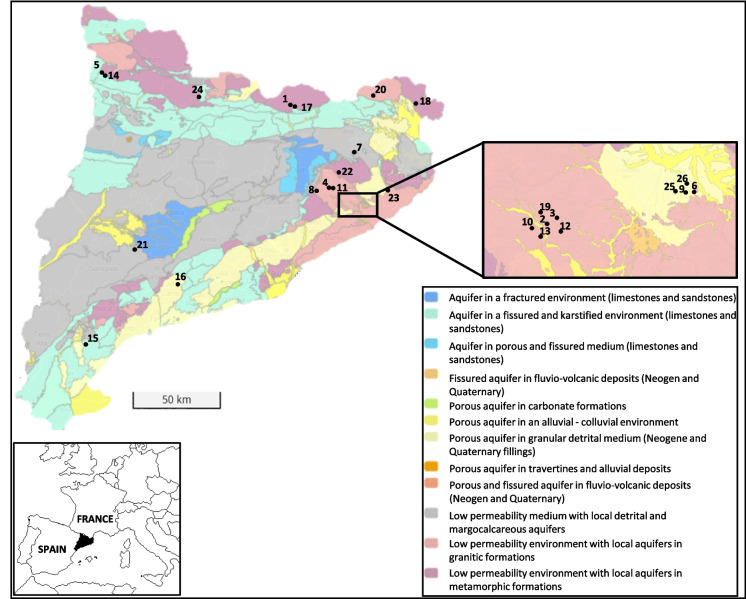
Map of Catalonia representing the aquifer units and locations of the origin of the samples (black dots) and the bottled mineral water samples represented by sample codes. Hydrogeological unit base map modified from Generalitat de Catalunya (2023)

Geologically, Catalonia presents a diverse range of lithological structures. Different hydrogeological units, formed by aquifer systems, are defined within these structures, as shown in Fig. 1. These aquifers comprise specific features in terms of hydraulics and rock composition which impart chemical components and mineralization to the groundwater.

Catalonia has a great variety of aquifer types, primarily classified based on their geological unit origins. Among them, three main geological units stand out: the Pyrenean Massif, the Catalan Coastal Ranges and the Catalan Central Basin, which are the sources of the natural mineral water bottled in Catalonia (Tapias et al., 2022). Table 1 presents all the natural mineral bottled water analysed in the present study, classified according to its geological unit origins. Consequently, they are grouped as granitic, metamorphic or sedimentary rocks based on the lithology of their location.

**Table 1:** Sample codes, aquifer type and lithology classification of natural mineral bottled water samples analysed in the present study

Catalan geological units	Sample code	Aquifer type (Generalitat de Catalunya, 2023)	Lithology according to Tapias et al. (2022)
Pyrenean Massif	1; 17	Low permeability media with local aquifers in the slates and granites of Núria-Canigó	Metamorphic rocks
	5; 14	- Aquifer system in the Paleozoic limestones, sandstones and slates of Boí-La Vall Fosca - Low permeability media with local aquifers in the slates of Sort-La Seu d’Urgell	
	24	- Aquifers of the Devorian limestones, sandstones and shales of Moixeró (Segre) - Low permeability environment with local aquifers in the Sort-La Seu d’Urgell slates	
Catalan Central Basin	15	Aquifer system in the Mesozoic limestones of the Móra depression	Sedimentary rocks
	16	Neogene and Quaternary detrital aquifer of Alt Camp	
	21	- Limestone aquifer of Tàrrega - Low permeability media with local aquifers in the limestones, marls and sandstones of Segrià-Garrigues	
Catalan Coastal Ranges	2; 3; 4; 8; 10; 11; 12; 13; 19	Low permeability media with aquifers in the granites of Montseny-Guilleries	Granitic rocks
	6; 9; 25; 26	- Low permeability media with local aquifers in the granites of the lower Costa Brava - Neogene detrital aquifer of la Selva - Alluvial aquifer of Onyar	
	7	Low permeability media with local aquifers in the Paleogene marls and sandstones of Garrotxa-Pla de l’Estany	Sedimentary rocks
	18	Low permeability media with local aquifers in the schists, slates and granites of Albera and Cadaqués	Granitic rocks
	20	Low permeability media with local aquifers in the granites and slates of La Jonquera and Roc de Frausa	
	22	Low permeability media with local aquifers in the schists and granites of Guilleries	
	23	Low permeability environment with local aquifers in the granites of the lower Costa Brava	

### Sample collection and radiochemical methods

Twenty-six samples of bottled natural mineral water (19 still waters and 7 sparkling waters) were analysed between January 2023 and November 2023. The origin locations of the aquifers for each sample are shown in Fig. 1.

All samples were directly purchased from supermarkets and online shops during the first quarter of 2023. The sampling covered nearly all of the natural mineral water brands listed in the Official Journal of the European Union, recognized in Catalonia and updated in February 2023 (Official Journal of the European Union, 2023; Secretaria de Salut Pública, 2023). However, one mineral water brand could not be analysed because it is no longer commercially available due to source closure.

Upon receipt at the laboratory, all samples were prepared and analysed. ^222^Rn activity was determined first, followed by non-radioactive parameters. Electrical conductivity (EC) was measured using a Cond70 + portable conductometer (XS Instruments, Italy), and pH was assessed using a pH meter (CRISON, Spain). The mineral composition (HCO_3_^−^, Ca^2+^, SiO_2_, Mg^2+^, Cl^−^, Na^+^, SO_4_^−2^) of the water samples was obtained from the labels or determined by our team when this information was unavailable. Subsequently, the radiological parameters were analysed, including gross alpha, gross beta, ^40^ K, ^210^Pb, ^210^Po, ^222^Rn, ^226^Ra, ^234^U and ^238^U. The procedures used for their determination are outlined in Table 2 and were previously described in a research article produced by members of our laboratory (Martínez et al., 2021). Minimum detectable activities (MDAs), calculated in accordance with the Currie definition (Currie, 1968), were used to estimate the method’s suitability for determining all of the radiological parameters outlined in Spanish RD 3/2023 (Ministerio de la Presidencia, 2023). These MDAs depend on the sample volume treated, the measuring times and the chemical yields and generally ranged between 5 and 60% lower than those established in Spanish legislation.

**Table 2:** Procedures, measurement techniques and MDA used to perform measurements of gross alpha, gross beta, ^210^Pb, ^210^Po, ^222^Rn, ^226^Ra, ^234^U and ^238^U

Radionuclide	Sample preparation	Sample volume (mL)	Measurement technique	Standard procedure	MDA (mBq/L)
Gross alpha	20 mL water, evaporation and deposition into a planchet	20	Solid scintillation counting	UNE-EN ISO 10704:2019	25
Gross beta	200 mL water, evaporation and deposition into a planchet	200	Proportional counter	UNE-EN ISO 10704:2019	40
^40^ K	50 mL water	50	Flame photometer (evaluated from stable potassium)	Internal method	0.25 ppm
^210^Pb	250 mL water, Sr resin, 4:16 mL sample/Ultima Gold™ LLT	250	Liquid scintillation counting	Internal method	15
^210^Po	500 mL water, iron hydroxide co-precipitation, autodeposition onto silver disc	500	Alpha spectrometry	Internal method	5
^222^Rn	10:10 mL water/Ultima Gold™ F	10	Liquid scintillation counting	ISO 13164–4:2015	1000
^226^Ra	500 mL water, barium/lead co-precipitation method	500	Solid scintillation counting	Internal method	2
^228^Ra	10 L water, evaporation until dry residue	–	Gamma spectrometry	Internal method	20
^234^U, ^238^U	500 mL water, UTEVA, electrodeposition onto stainless steel disc	500	Alpha spectrometry	ISO 13166	1

The radioanalytical procedures employed to determine gross alpha, gross beta, ^210^Pb, ^210^Po, ^222^Rn, ^226^Ra, ^234^U and ^238^U in water samples were externally validated through our laboratory’s participation in national and international proficiency tests at regular intervals. The Z-scores, a standard measure of laboratory systematic error calculated using the certified value and standard deviation of the exercise, ranged between − 1 and 1. Given that the acceptance criteria for this test require Z-scores to fall between − 2 and + 2, these results demonstrate the robustness and suitability of all methods for their intended purposes.

### Radiation dose assessment

The total annual committed effective dose was evaluated for the samples that did not meet the parameters stipulated in Spanish legislation (gross alpha < 0.1 Bq/L, gross beta < 1 Bq/L). In those cases, annual effective dose (AED), in µSv/year, was calculated for infants, children and adults using to the following equation:

1 $$\text{AED}=\sum\nolimits_{i=1}^{n}{A}_{\text{w}}\times {I}_{\text{w}}\times {\text{IF}}_{\text{w}}$$

where *A*_w_ represents the measured activity of individual radionuclides (Bq/L) and *I*_w_ is the estimated water consumption for a human over 1 year of age (150 L for infants, 350 L for children and 730 L for adults, respectively) (United Nations Scientific Committee on the Effects of Atomic Radiation, 2008). The ingestion effective dose coefficient factor (IF_w_) values (Sv/Bq) used in the calculations for ^210^Pb, ^210^Po, ^226^Ra, ^234^U and ^238^U were taken from the International Commission on Radiological Protection (International Comission on Radiological Protection, 1996).

Additionally, lifetime cancer risk (LCR) was calculated using the following equation:

2 $$\text{LCR}=\text{AED}\times \text{DL}\times \text{RF}$$

where DL represents the lifespan (79 years), and RF is the risk factor (Sv^−1^), which is 0.05.

### Data handling and statistics

All data were managed using Microsoft Office 365 Excel®. Statistical analyses were conducted using Matlab 2023a (MathWorks Inc., Natick, MA, USA) and PLS Toolbox 9.3 (Eigenvector Inc., Manson, WA, USA) for Matlab.

Principal component analysis (PCA) was used for a multivariate analysis of the autoscaled chemical data in order to identify any relationships among the physicochemical, radiological and lithological parameters. This technique graphically represents not the original variables, but rather new variables termed principal components (PCs), which are a linear combination of the original variables, and follow the direction of maximum variation in the data.

For all quantitative assessments, concentrations below the detection limit were considered as half of the detection limit (MDA/2) for statistical analysis (U.S. EPA, 2006).

## Results and discussion

Catalonia exhibits a complex hydrogeological and lithological composition, which could influence the radiological content of groundwater samples sold as bottled natural mineral water.

In this section, we present the physicochemical parameters and gross alpha and gross beta activities, both used as screening parameters to assess the annual committed effective dose according to RD 3/2023 (from which NMW is exempt), determined in 26 natural mineral waters and relate them to their hydrogeological units of origin. For water samples exceeding gross alpha or gross beta, the activity of certain natural radionuclides such as ^210^Pb, ^210^Po, ^226^Ra, ^234^U and ^238^U was determined to assess the potential health risk associated with ingestion of the water.

### Gross alpha and gross beta activity concentrations

**Fig. 2:**
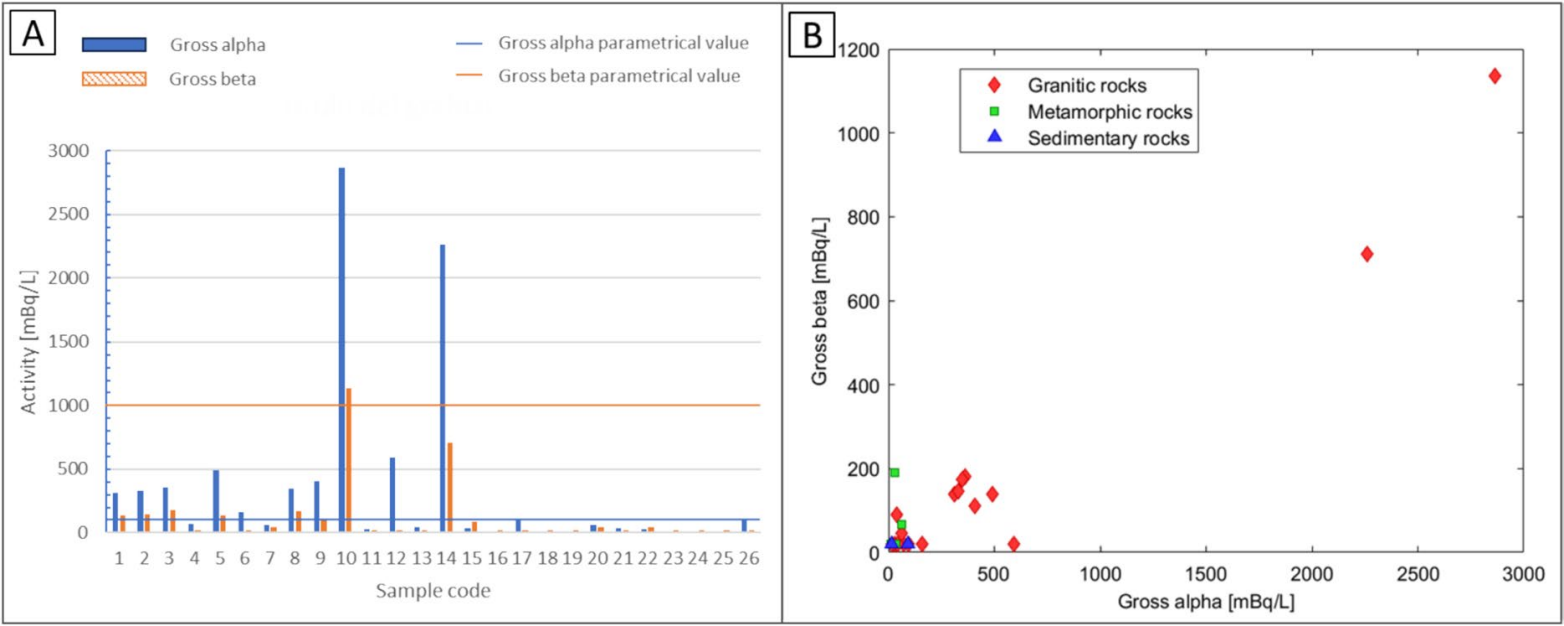
**A** Gross alpha and gross beta activity concentrations of 26 NMW samples (*y*-axis in 10-base logarithmic scale). **B** Gross alpha and gross beta related with rock composition

Gross alpha and gross beta activity concentrations are shown in Fig. 2A. Gross alpha activities ranged between < MDA and 2865 ± 200 mBq/L, with an average of approximately 500 mBq/L in NMW samples from granitic aquifers; 38.5% of samples (samples with codes 2, 3, 4, 8, 9, 11, 12, 13, 19 and 22) exceeded the Spanish parametric value for gross alpha (100 mBq/L). As shown in Fig. 2B, their high alpha activity is linked to lithologic compositions mainly dominated by granitic aquifers from Girona Province. It is noteworthy that some NMW samples from the same type of aquifer did not exceed the gross alpha parametric value. Despite their granitic aquifer origin, the radiological characteristics of the samples may also be influenced by other factors, such as distance and time travelled underground before sampling, which could affect the reaction time with rocks as well as their mineral composition. Our data are consistent with previous studies in which water samples with high alpha activity concentrations originated from granitic geological areas (Martínez et al., 2021; Pérez-Moreno et al., 2020).

Gross beta activity (without ^40^ K) ranged between < MDA and 1135 mBq/L. Only one sample (sample 13) exceeded the parametric value. Beta emitters from the ^238^U disintegration chain, which are present with high activity in this sample, are the main contributors to gross beta. NMW samples from metamorphic and sedimentary aquifers presented gross alpha and gross beta activities below their respective parametric values.

**Fig. 3:**
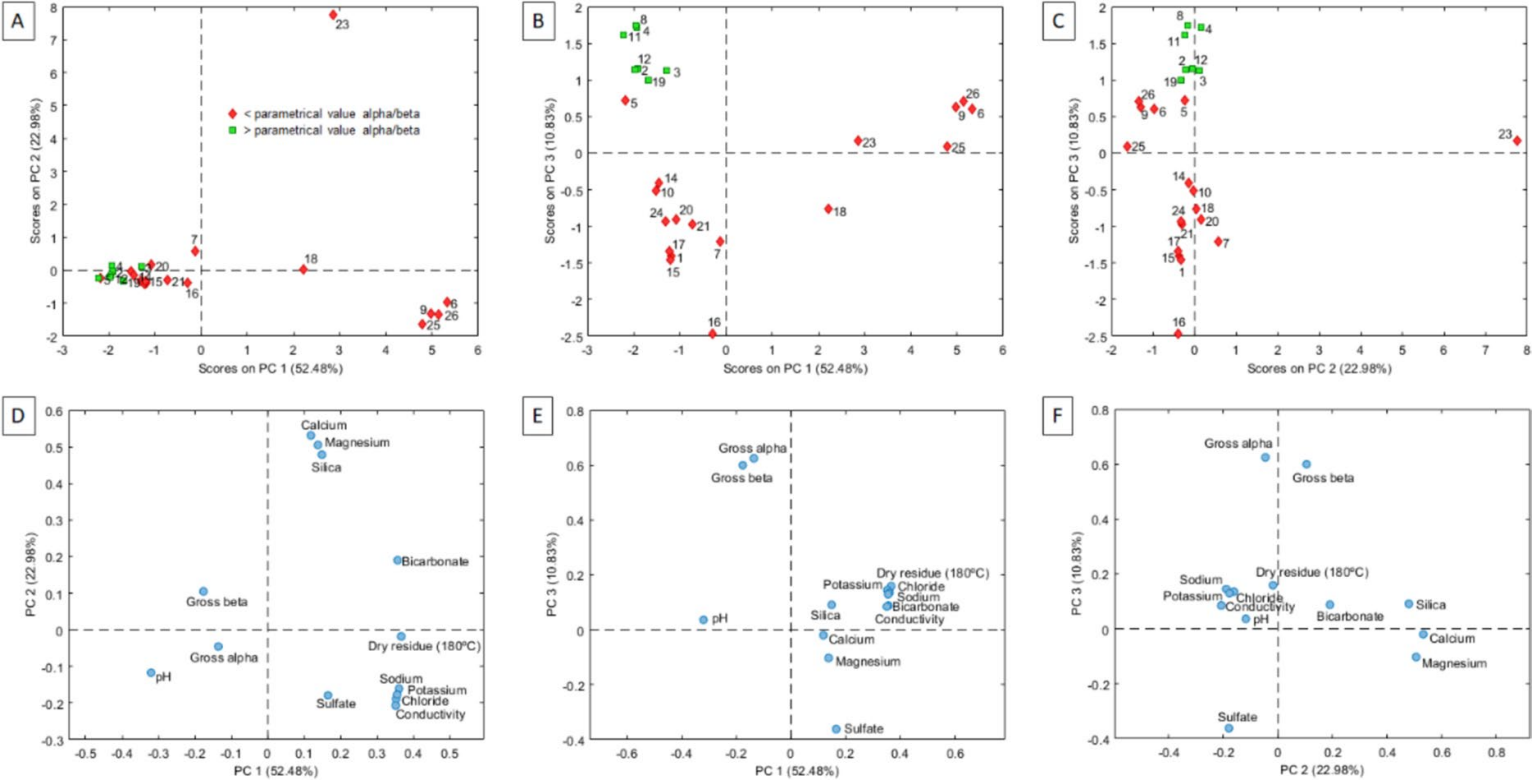
PCA results score plot (**A**–**C**) and loadings (**D**–**F**) for the first three PCs of the PCA

A principal component analysis was performed to summarize and elucidate the relationships between physicochemical and gross alpha and gross beta parameters in all the NMW samples tested. As shown in Fig. 3, three PCs explained 86% of the total variance. The first PC (PC1), representing 52.48% of the total variance, is significantly correlated with dry residue, bicarbonate, chloride, sodium, potassium and conductivity (positive correlation) as well as pH (negative correlation). The second PC (PC2) accounts for 22.98% of the total variance and is significantly correlated with calcium, silica and magnesium (positive correlation) and chloride, sodium, sulfate, potassium and conductivity (negative correlation). Interestingly, the third PC (PC3) exhibits a notable correlation with gross alpha and gross beta (positive correlation). Consequently, samples positioned in the positive values of PC3 in the score plot tend to have higher values of gross alpha and gross beta. This observation is evident in Fig. 3B and C, where all samples exceeding the gross alpha and beta parametric values are grouped in the positive values of PC3. Generally, samples exceeding the gross alpha and beta parametric values tend to be characterized by high pH values and low values of all or most of the other physicochemical parameters. However, there were exceptions and not all samples with similar physicochemical composition necessarily possessed high radioactivity content.

Data on 12 physicochemical parameters was collected from the commercial labels of the natural mineral water studied or identified through our own analysis. In general, the major ion composition, which determines the chemical character of each sample, varies widely in concentration among the 26 analysed NMW samples with calcium-bicarbonate and sodium-bicarbonate water types comprising the largest groups. This characteristic mineralization depends directly on the geology of the spring location and other aquifer features like residence time and depth. As an example, samples from the Montseny-Guilleries Massif, which circulated through granitic rocks, exhibited a characteristic composition, with bicarbonate as the dominant anion and a relatively low chloride content and a high silica content. On the other hand, sparkling waters associated with thermal springs (samples with codes 6, 9, 25 and 26), located predominantly in granitic rocks but also in other lithologies like volcanic or metamorphic stones, had higher mineralization, as shown in Fig. 3A (these samples are the opposite of those with high gross alpha/beta values, and therefore, as previously mentioned, they tend to have higher mineralization).

Finally, a correlation matrix was performed to detect correlations between chemical components and gross alpha and beta activities. As shown in Table 3, one set of variables yielded high correlation coefficients (*r* > 0.75), including dry residue, conductivity, bicarbonate, chloride, sodium and potassium, with gross alpha and gross beta presenting the highest correlation. Correlation was not determined for gross beta and potassium because, in this case, gross beta was considered without the contribution of ^40^ K. In the present study, natural radioactivity, expressed as the two screening parameters, did not depend primarily on the salt content of the analysed waters, indicating that NMW with high salinity is not those with higher gross alpha activity. Radionuclide release from the solid to liquid phase should be considered as a gross alpha source, including parameters like the surface roughness of the rock matrices and the irregular distribution of minerals on the surface of the rocks, among others (Bonotto et al., 2009).

**Table 3:** Spearman correlation matrix (significant correlations at significance level *p* < 0.05)

	Gross alpha	Gross beta	Dry residue	Bicarbonate	Calcium	Silica	Magnesium	Chloride	Sodium	Sulfate	pH	Conductivity	Potassium
Gross alpha	1												
Gross beta	*0.96*	1											
Dry residue	− 0.12	− 0.16	1										
Bicarbonate	− 0.18	− 0.19	*0.90*	1									
Calcium	0.06	0.07	0.28	0.56	1								
Silica	− 0.12	− 0.08	0.35	0.64	*0.77*	1							
Magnesium	0.17	0.15	0.29	0.52	*0.91*	0.62	1						
Chloride	− 0.04	− 0.10	0.92	*0.76*	0.04	0.10	0.09	1					
Sodium	− 0.17	− 0.20	*0.93*	*0.81*	0.02	0.16	0.04	*0.97*	1				
Sulfate	0.45	0.33	0.20	0.08	0.19	− 0.18	0.50	0.32	0.12	1			
pH	0.25	0.25	− 0.74	* − 0.81*	− 0.35	− 0.44	− 0.33	− 0.63	− 0.70	0.04	1		
Conductivity	− 0.20	− 0.23	*0.89*	*0.75*	− 0.07	0.09	− 0.04	*0.95*	*0.98*	0.10	− 0.70	1	
Potassium	− 0.20	− 0.23	*0.91*	*0.79*	− 0.01	0.15	− 0.01	*0.96*	*0.99*	0.08	− 0.69	*0.99*	1

### Naturally occurring radionuclides in bottled water

**Fig. 4:**
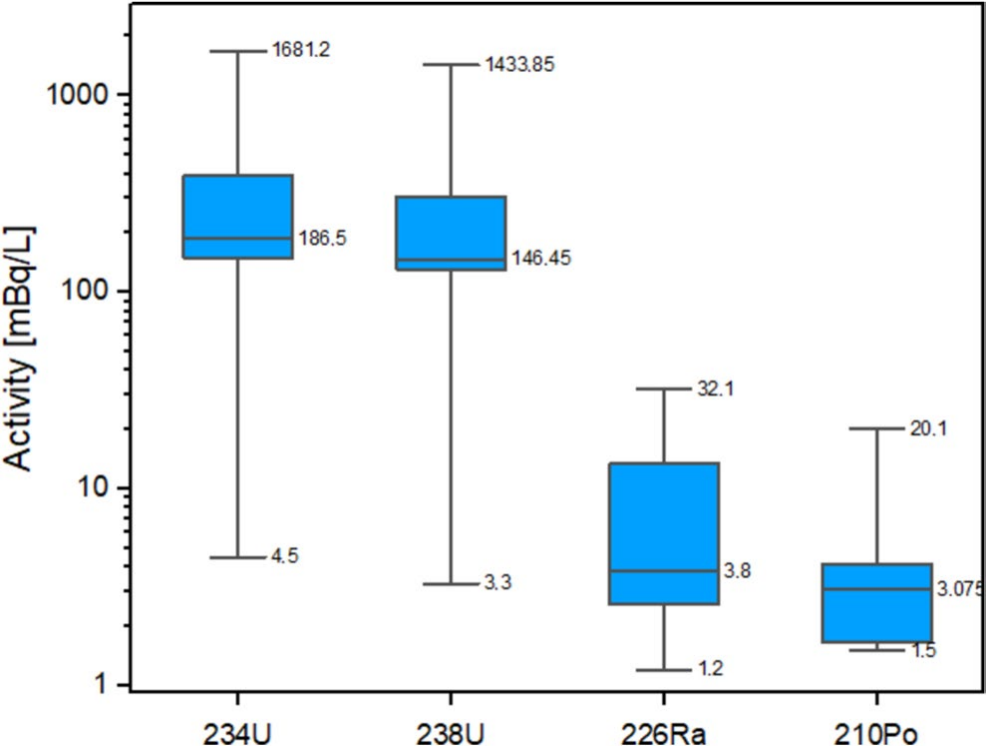
Box and whisker plot of natural radionuclide activity obtained for samples exceeding the alpha parametric value. Lower and upper box edges correspond to the 25% and 75% percentile, respectively. The central box line is the median, and the whiskers correspond to the minimum and maximum activity concentrations

The activities of natural radionuclides such as ^234^U, ^238^U, ^226^Ra, ^210^Pb and ^210^Po in NMW samples exceeding the gross alpha parametric value (samples 2, 3, 4, 8, 9, 11, 12, 13, 19 and 22) are shown in Fig. 4 and Table 4. In general, and as can be seen in Fig. 4, considering the median values, the radionuclides ^234^U and ^238^U are the greatest contributors to gross alpha activity, followed by ^226^Ra and ^210^Po. This radionuclide contribution aligns with results obtained from the characterization of NMW samples in granitic areas from Sweden (0.2–714 mBq/L of ^238^U and 0.2–1162 mBq/L of ^234^U) (Piñero-García et al., 2021). In our study, all these samples correspond to areas with predominantly granitic formations in Girona Province, where rocks are known to contain high levels of ^238^U disintegration chain radionuclides (Piñero-García et al., 2021).

**Table 4:** Activities (mBq/L) of ^234,238^U, ^226^Ra, ^210^Po and ^210^Pb, and activity ratios of ^234^U/^238^U, ^226^Ra/^234^U, ^210^Pb/^226^Ra and ^210^Po/^226^Ra

Sample code	^234^U (mBq/L)	^238^U (mBq/L)	^226^Ra (mBq/L)	^210^Po (mBq/L)	^210^Pb (mBq/L)	^234^U/^238^U	^210^Pb/^226^Ra	^210^Po/^226^Ra
2	131 ± 8	99 ± 6	3.8 ± 0.7	1.6 ± 0.7	–	1.3	–	0.4
3	149 ± 9	142 ± 9	3.8 ± 0.9	4 ± 1	–	1.1	–	0.9
4	150 ± 9	136 ± 8	4 ± 1	3 ± 1	–	1.1	–	0.8
8	169 ± 10	131 ± 8	2.9 ± 0.7	2 ± 1	–	1.3	–	0.7
9	4.5 ± 0.9	3.3 ± 0.7	32 ± 2	1.5 ± 0.8	–	1.4	–	0.0
11	204 ± 12	151 ± 9	2.6 ± 0.6	3 ± 1	–	1.3	–	1.1
12	209 ± 13	157 ± 10	1.7 ± 0.6	1.7 ± 0.8	–	1.3	–	1.0
13	1680 ± 87	1434 ± 75	13 ± 1	20 ± 4	104 ± 35	1.2	7.8	1.5
19	390 ± 22	307 ± 18	1.2 ± 0.6	4 ± 1	–	1.3	–	3.5
22	1275 ± 61	1112 ± 59	17 ± 2	19 ± 4	90 ± 35	1.1	5.3	1.1

As Table 4 shows, ^234^U and ^238^U activities were in the range of 4.5 to 1681.2 mBq/L and 3.3 to 1433.9 mBq/L, respectively, in these NMW samples, indicating high variability despite a common origin (granitic aquifers). As stated in the literature, this great variability in activity can be attributed to the geology of the water source, among other factors such as adsorption/desorption processes in the water system and residence time (International Atomic Energy Agency, 2023). High uranium activity was determined in the samples coded 13 and 22, probably related to the enhancement of uranium leaching from host rock due to specific aquifer properties and the mineralogical composition of the granitic formations (Borrego-Alonso et al., 2023). The ^234^U/^238^U ratio ranged from 1.1 to 1.4 in these ten NMW, indicating a weak disequilibrium between the two uranium isotopes due to processes such as preferential ^234^U leaching caused by alpha recoil effects, among other physicochemical processes (Dinh Chau et al., 2011).

The activity of ^226^Ra ranged from 1.2 to 32.1 mBq/L in granitic aquifers, which is similar to the values obtained by Pérez-Moreno et al. (2020) in Spanish areas where granitic formations are predominant (1.1–196 mBq/L of ^226^Ra). In general, ^226^Ra activities were below 100 mBq/L. These results are in line with the behaviour of this isotope in European water systems where low activities are associated with a relatively high uranium content (higher than 100 mBq/L) (Dinh Chau et al., 2011; Ortega et al., 1997). This could be characteristic of young groundwaters where ^238^U remains in solution while ^226^Ra has not reached its secular equilibrium in the rock-water system.

^228^Ra activity co-occurred in the same natural mineral waters with higher ^226^Ra, although they originate from different decay chains. ^228^Ra activities were below 20 mBq/L (the MDA) in samples 13 and 22.

Samples with high ^226^Ra activities also exhibited ^210^Pb, as in the case of samples 13 and 22. This trend has been observed previously in other Catalan mineral waters from nearby areas (Ortega et al., 1997), specifically in thermal bottled water, with a high concentration of ^226^Ra, 860–960 mBq/L, and a high concentration of ^210^Pb, 200–1130 mBq/L. Neither isotope is in equilibrium, likely due to ^210^Pb originating from ^222^Rn, which has a high diffusion rate into groundwater aquifers, or because of high ^210^Pb desorption rates (Molla et al., 2021; Pérez-Moreno et al., 2020). In this context, an activity of 1150.0 mBq/L of ^222^Rn was determined in the sample coded 13, calculated at the time of measurement due to the unavailability of bottling time information. In the remaining samples, this radionuclide activities were below the MDA of 1000 mBq/L.

Low levels of ^210^Po were detected in bottled water, with an average value of 6.0 ± 1.6 mBq/L. In all cases, the value was lower than the derived concentration for this isotope (100 mBq/L). As shown in Table 4, samples 13 and 22 presented the highest ^210^Po activities due to their high uranium content. These results are consistent with ^210^Po activity in groundwater reported in the literature (Kinahan et al., 2020; Piñero-García et al., 2021; Slavchev et al., 2022). For example, in bottled water samples from Sweden, the ^210^Po content is 0.4–115 mBq/L (Piñero-García et al., 2021); from Japan, it is 1.0–4.9 mBq/L (Kinahan et al., 2020); and from Bulgaria, it is < 0.3–13.6 mBq/L. In five samples, ^210^Po/^226^Ra exhibited a deficiency of ^210^Po, probably due to its high sorption affinity with colloidal mineral particles. However, as with the case of ^210^Pb/^226^Ra, the other samples had higher related ratios, which is consistent with the arguments given above.

### Estimation of annual effective dose and risk assessment

We conducted a radiological health assessment in light of the results derived from the radiological characterization performed on the 26 natural mineral water samples.

Gross alpha and gross beta activities were used as screening levels to check the parametric value of the indicative dose (ID), which is set at 100 µSv/year by Spanish legislation (Ministerio de la Presidencia, 2023), although NMW is exempt from these requirements. Therefore, if gross alpha and gross beta activities do not exceed 100 mBq/L and 1000 mBq/L, respectively, the ID can be considered lower than 100 µSv/year, and no further radiological study is required. This was the case of all the natural mineral waters analysed, except for samples 2, 3, 4, 8, 9, 11, 12, 13, 19 and 22, which, based on the provided information, are suitable for human consumption.

On the other hand, the gross alpha screening level was exceeded in 38% of the samples. In these samples, we evaluated the AED due to the ingestion of natural radionuclides (^210^Pb, ^210^Po, ^226^Ra, ^234^U and ^238^U) present in bottled mineral water available on the Catalan market. The samples with codes 2, 3, 4, 8, 9, 11, 12 and 19 exhibited good radiological quality, as their calculated AEDs ranged from 2.2 to 27.4 µSv/year with an average value of 13.6 µSv/year for adults, from 2.5 to 21.3 µSv/year with an average value of 11.0 µSv/year for children and from 8.5 to 53.9 µSv/year with an average value of 29.0 for infants. All these values are below the recommended AED (100 µSv/year), but they account for larger doses for children and infants than for adults. This is due to the fact that even though children and infants consume less water, their corresponding effective dose coefficients are higher than those for adults, resulting in a higher AED. The contribution of each natural radionuclide to the averaged AED for the previously mentioned samples was also assessed, and the results are shown in Table 5. Uranium isotopes were responsible for the highest average contribution to AED for the adult and children’s groups because they exhibit the highest activities in the analysed bottled waters. However, ^210^Po is the primary contributor to the AED for the infants’ group, despite its low activity. This is related to its high effective dose coefficient due to its high radiotoxicity (University of Michigan: Environment Health & Safety, 2016).

**Table 5:** Contribution (average values), in %, of ^210^Po, ^226^Ra, ^234^U and ^238^U to the AED

	AED (µSv/year)	^210^Po (%)	^226^Ra (%)	^234^U (%)	^238^U (%)
Infant	29.0	35.0	7.1	27.7	30.2
Children	11.0	21.5	6.1	34.0	37.1
Adult	13.6	16.8	4.4	38.2	40.7

**Fig. 5:**
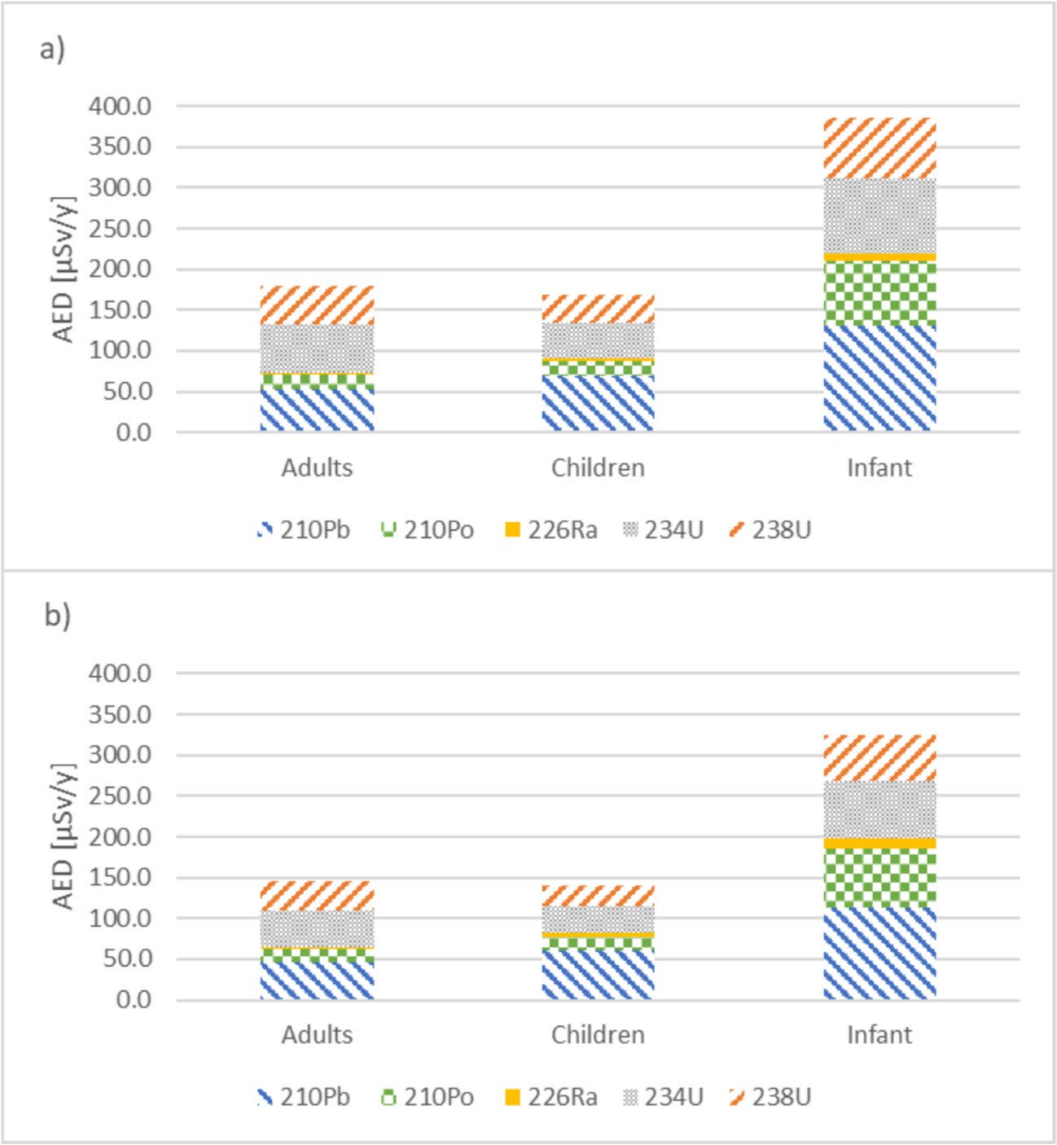
AED, in µSv/year, due to sample 13 (**a**) and sample 22 (**b**) water consumption for infants, children and adults and individual radionuclide contribution

**Fig. 6:**
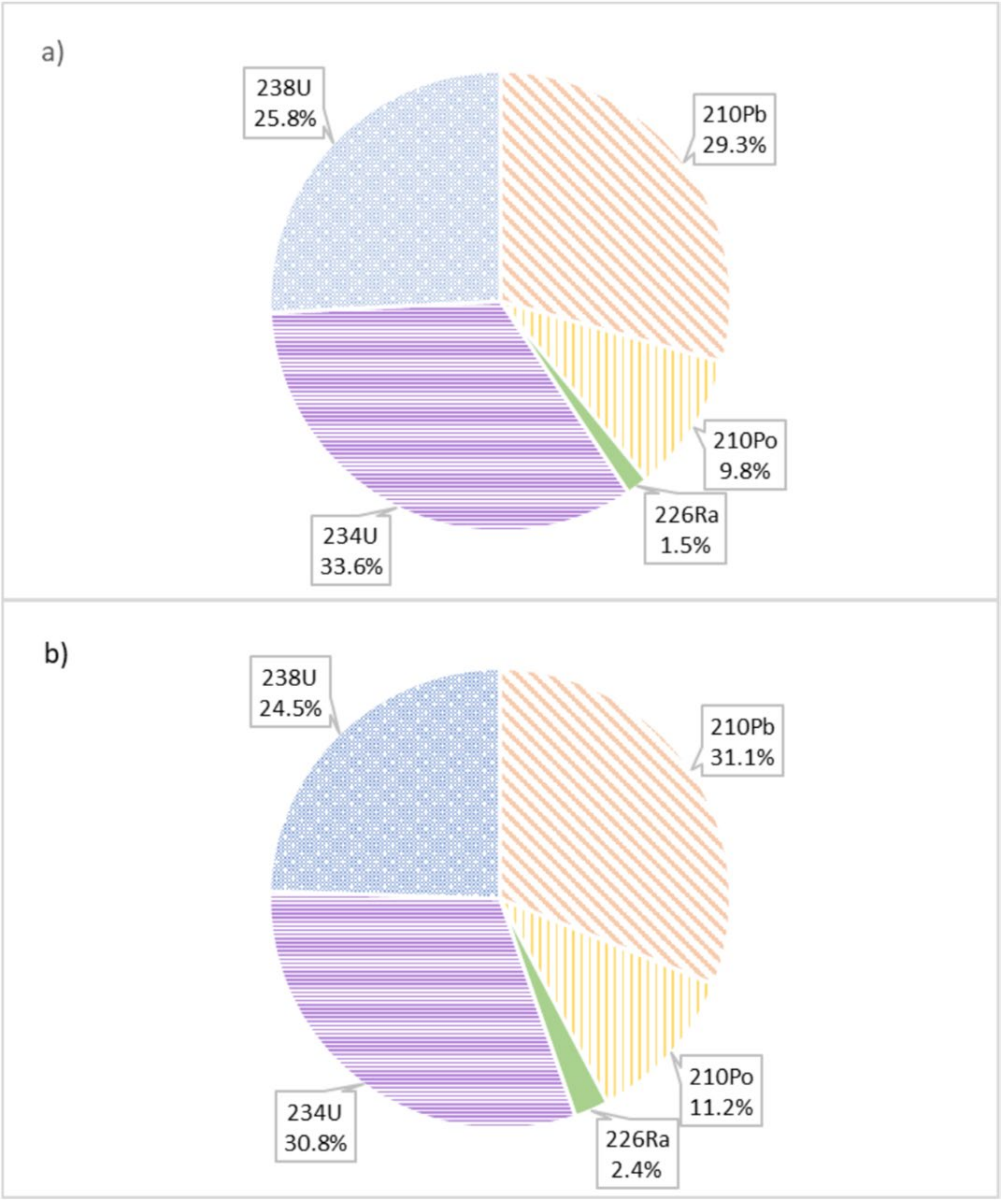
Individual radionuclide contribution (%) in AED due to sample 13 (**a**) and sample 22 (**b**) water consumption for adults

Among the 26 bottled natural mineral water samples analysed in the present study, two of them (samples 13 and 22) exceeded the AED (100 µSv/year) parametric value for each specific age category, as shown in Fig. 5. Although the volume of water consumed increases from infants to adults (150 L > 350 L > 730 L), larger doses are associated with children and infants due to age-dependent coefficient dose factors, considering metabolism rates for different radionuclides and age groups. Adults, children and infants follow the same trend, with the AED dominated by ^210^Pb and ^234^U, which account for over 30% and 32% of ingestion dose, respectively, as shown in Fig. 6. Although the activities of ^210^Pb are lower compared to those of uranium isotopes, the dose for lead is higher due to its higher committed effective dose per unit intake. The next most significant contributor is ^238^U (25%), followed by ^210^Po (11%) and ^226^Ra (2%).

The evaluation of the AED and the LCR due to the intake of natural radionuclides from samples 13 and 22 was conducted using average activity values of individual radionuclides determined in two different batches purchased 6 months apart. Additionally, LCR and AED parameters were estimated for adults in two scenarios. In the first scenario, the AED was assessed considering an annual water consumption per person of 730 L/year, which is the value defined in Spanish legislation (Ministerio de la Presidencia, 2023). In the second scenario, the 2022 annual consumption of NMW water per person (132 L/year) was taken to estimate the AED (Asociación Aguas Minerales de España, 2022). As Table 6 shows, AEDs from the second scenario are significantly lower than the recommended AED parametric value set by Spanish legislation and account for 30% of the UNSCEAR estimated ingestion dose of approximately 300 µSv/year from food and drinking water (United Nations Scientific Committee on the Effects of Atomic Radiation, 2008). However, in the first scenario, AED exceeds 100 µSv/year due to the higher volume of water consumed. Unlike the other samples, samples 13 and 22 contain ^210^Pb (Fig. 6). In these cases, the high dose levels received via ingestion are a consequence of the presence of lead and uranium radionuclides at high activities. The ^210^Pb present in both samples is also considered a highly toxic radionuclide with a high effective dose coefficient, contributing to the increase in AED (University of Michigan: Environment Health & Safety, 2016). Finally, the LCR results obtained in both scenarios (Table 6) are lower than 10^−3^, the acceptable lifetime cancer risk (Altlkulaç et al., 2022).

**Table 6:** Annual effective dose and life cancer risk of sample codes 13 and 22, evaluated for two scenarios

Scenario	Sample code	AED (µSv/year)	LCR
First (730 L/year)	13	179.0	7.1E − 04
	22	145.9	5.8E − 04
Second (132 L/year)	13	32.4	1.3E − 04
	22	26.4	1.0E − 04

To summarize, AED combined with LCR provides enough information to conclude that there is no radiological risk associated to the consumption of water samples 13 and 22. In this study, the health risk assessment (according to RD 3/2023 requirements) due to the ingestion of NMW was conducted in two batches of water samples to evaluate the radiological content variability due to seasonality. It is worth noting that there has been a lack of rainfall in the studied area in the past year due to climate change, which may have affected the aquifers recharge conditions, possibly leading to temporary variations in mineralization and natural radiological content in water at the source (Dinh Chau et al., 2011). Therefore, periodic radiological water surveillance assessments are recommended in order to detect the temporal variability of natural radiological content and provide the basis for possible remedial actions in terms of radiation protection and to gain a radiological perspective with regard to dose due to NMW ingestion.

## Conclusions

This study provides detailed results on the determination of the radiological content and some of the physicochemical values of bottled natural mineral water in Catalonia. Eighty percent of natural mineral water in Catalonia originates from granitic aquifers (Girona). The most relevant finding is that these samples were characterized by high gross alpha activity, exceeding 100 mBq/L, mainly due to the contribution of uranium radioisotopes. Occasionally, activities of more than 1000 mBq/L of ^234^U and ^238^U as well as ^210^Pb were detected in two samples from this area. Despite the statistical analysis, no pattern could be established, and not all samples with high pH and low values of sulfate, sodium, chloride, etc. necessarily had a high radioactivity content.

The total annual effective dose calculated by ingestion for adults was below the AED parametric value stipulated in RD 3/2023 (100 µSv/year) in 92% of the natural mineral water samples analysed in this study. For the children and infant groups, although the AED was also below 100 µSv/year, it was higher than for adults. Eight percent of NMW samples exceeded the AED parametric value. In these situations, AED value ranged between 325 and 385 µSv/year for infants, between 141 and 169 µSv/year for children and between 146 and 179 µSv/year for adults. The main contributors to these AED values are ^234,238^U > ^210^Pb > ^210^Po > ^226^Ra. However, after combining AED with life cancer risk assessment, we concluded that there was no significant risk associated with the consumption of these two brands of natural mineral waters, although surveillance assessments in relation to radiation protection is advisable.

## Data Availability

No datasets were generated or analysed during the current study.
